# Determination of Interleukin 27-Producing CD4^+^ and CD8^+^ T Cells for The Differentiation Between Tuberculous and Malignant Pleural Effusions

**DOI:** 10.1038/srep19424

**Published:** 2016-01-19

**Authors:** Ya-Lan Liu, Yan-Bing Wu, Kan Zhai, Xiao-Juan Wang, Huan-Zhong Shi

**Affiliations:** 1 Department of Respiratory and Critical Care Medicine, Beijing Institute of Respiratory Medicine and Beijing Chao-Yang Hospital, Capital Medical University, Beijing, China

## Abstract

The numbers of IL-27^+^ CD4^+^ and IL-27^+^ CD8^+^ T cells have been found to be increased in tuberculous pleural effusion (TPE) as compared with malignant pleural effusion (MPE). The objective of the present study was to investigate whether pleural IL-27^+^ CD4^+^ and IL-27^+^ CD8^+^ T cells can distinguish patients with TPE from those with MPE. Paired specimen of pleural fluid and peripheral blood were collected from 35 patients with TPE and 46 MPE. The numbers of IL-27^+^ CD4^+^ and IL-27^+^ CD8^+^ T cells were simultaneously determined by flow cytometry. Receiver operating characteristic curve analysis was used to evaluate the capacity of IL-27^+^ CD4^+^ and IL-27^+^ CD8^+^ T cells to differentiate TPE from MPE. The sensitivity, specificity, positive likelihood ratio (PLR), negative likelihood ratio (NLR), positive predictive value (PPV), and negative predictive value (NPV) of IL-27^+^ CD4^+^ T cells were 94.3%, 93.5%, 14.46, 0.06, 91.7%, and 95.6%, respectively. The sensitivity, specificity, PLR, NLR, PPV and NPV of IL-27^+^ CD8^+^ T cells were 80.0%, 93.5%, 12.27, 0.21, 90.3% and 86.0%, respectively. The number of IL-27^+^ CD4^+^ in pleural fluid is a helpful diagnostic biomarker for the diagnosis of TPE, which performs better than that of IL-27^+^ CD8^+^ T cells.

Both tuberculous pleural effusion (TPE) and malignant pleural effusion (MPE) are lymphocytic pleural effusions. Differential diagnosis of TPE from pleural effusions with the other etiologies, especially MPE, is always a diagnostic challenge^1^,^2^. The most accurate procedure for diagnosing TPE is medical thoracoscopy (specificity 100%, sensitivity 98–100%); however, medical thoracoscopy is an invasive technique associated with major (1.8%) and minor (7.3%) complications. Where diagnostic difficulty exists, a number of biomarkers in pleural fluid, including IFN-γ^3^ and adenosine deaminase (ADA)^4^, have been proposed for the diagnostic purpose.

In 2012, we reported for the first time that the concentration of IL-27 in TPE is significantly higher than that in non-TPEs, and that IL-27 had a sensitivity of 92.7% and a specificity of 98.4% for distinguishing TPE from MPE^5^. Our another study further revealed that the diagnostic efficiency of IL-27 was better than that of IFN-γ or ADA, and that the combinations of IL-27, IFN-γ or/and ADA measurements provide even higher diagnostic accuracy for discriminating TPE from non-TPEs^6^. The subsequent studies also confirmed that elevated IL-27 levels are present in TPE, and the product of IL-27 and ADA can improve the sensitivity of ADA in the diagnostic approach to TPE^7^,^8^.

Our previous study also showed that the numbers of IL-27^+^ CD4^+^ and IL-27^+^ CD8^+^ T cells in TPE were significantly higher than their corresponding counterparts in MPE. In the present study, we have extended our previous studies and explored the diagnostic performance of pleural IL-27^+^ CD4^+^ and IL-27^+^ CD8^+^ T cells in distinguishing TPE from MPE.

## Results

### Biochemical and cytological characteristics in pleural effusions

**Figure 1:**
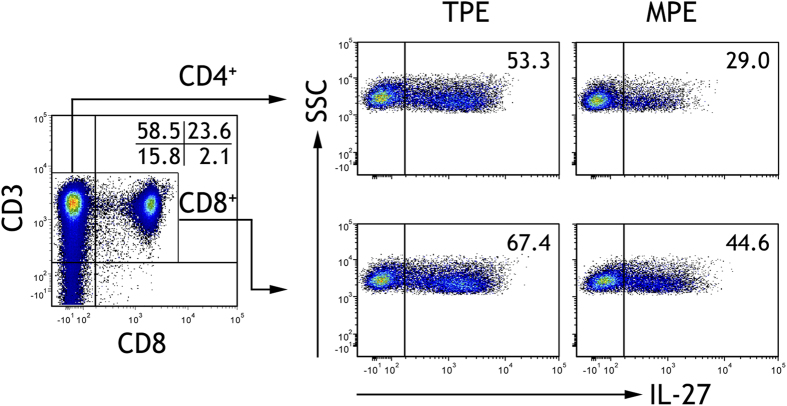
The representative flow cytometric dot plots showing expressions of IL-27 on CD4^+^ T cells in tuberculous pleural effusion (TPE) and malignant pleural effusion (MPE).

Some demographic data of patients studied, as well as biochemical and cytological characteristics in TPE and MPE are illustrated in Table 1 and Fig. 1. As expected, patients with TPE were much younger than patients with MPE (p = 0.002). The concentrations of protein, glucose, and lactate dehydrogenase in TPE were not different from those in MPE (all p > 0.05). Consistent with our previous findings, the numbers of nucleated cells in TPE were higher than those in MPE (p = 0.038), and large percentages of these cells were lymphocytes, with some neutrophils, macrophages, and mesothelial cells. In addition, malignant cells could be found in 22 of 46 patients with MPE.

### Numbers of IL-27^+^ CD4^+^ and IL-27^+^ CD8^+^ T cells in pleural effusions

**Figure 2:**
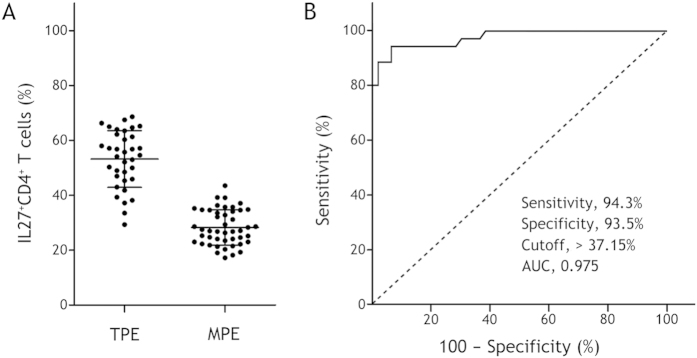
IL-27^+^ CD4^+^ T cell numbers in tuberculous pleural effusion (TPE) and malignant pleural effusion (MPE). Panel A shows the comparison of IL-27^+^ CD4^+^ T cell numbers in TPE and those in MPE. Horizontal bars indicate means ± SDs. Comparisons of data between TPE and MPE were performed using Student’s *t* test. Panel B shows the receiver operating characteristic curve of IL-27^+^ CD4^+^ T cells for 35 TPE patients versus 46 MPE patients.

**Figure 3:**
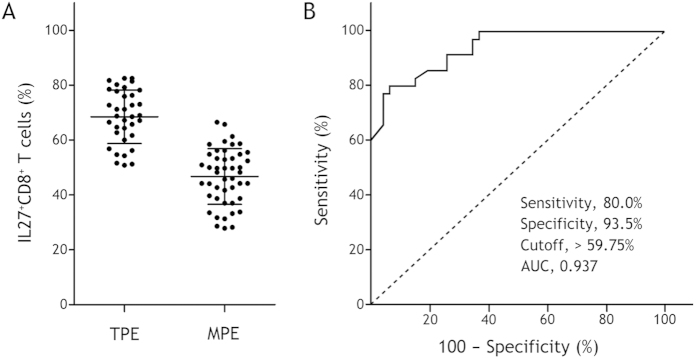
IL-27^+^ CD8^+^ T cell numbers in tuberculous pleural effusion (TPE) and malignant pleural effusion (MPE). Panel A shows the comparison of IL-27^+^ CD8^+^ T cell numbers in TPE and those in MPE. Horizontal bars indicate means ± SDs. Comparisons of data between TPE and MPE were performed using Student’s *t* test. Panel B shows the receiver operating characteristic curve of IL-27^+^ CD8^+^ T cells for 35 TPE patients versus 46 MPE patients.

As shown in Table 2, the numbers of IL-27^+^ CD4^+^ (Fig. 2A) and IL-27^+^ CD8^+^ (Fig. 3A) T cells in TPE were significantly higher than their corresponding counterparts in MPE (95% CIs for the differences were 21.2% to 28.6% and 17.3% to 26.2%, respectively; both p < 0.001). The numbers of these IL-27^+^ T cells were much higher in TPE than those in the corresponding blood (95% CIs for the differences were 21.2 to 25.7% and 21.1 to 27.3%, respectively; both p < 0.001).In addition, the numbers of IL-27^+^ CD4^+^ T cells in MPE were not different from those in blood (95% CI for the difference was −2.2 to 1.3%; p = 0.601), while the numbers of IL-27^+^ CD8^+^ T cells in MPE were higher than those in blood (95% CI for the difference was 0.39 to 5.8%; p = 0.026).

### Diagnostic values of pleural IL-27^+^ CD4^+^ and IL-27^+^ CD8^+^ T cells

The AUC for IL-27^+^ CD4^+^ T cells was 0.975. A cutoff value of 37.15% had the highest accuracy (minimal false negative and false positive results) for TPE detection (sensitivity, 94.3%; specificity, 93.5%) (Fig. 2B). The AUC for IL-27^+^ CD8^+^ T cells was 0.937 at a cutoff level of 59.75% (sensitivity, 80.0%; specificity, 93.5%) (Fig. 3B).

The diagnostic measures of pleural IL-27^+^ CD4^+^ and IL-27^+^ CD8^+^ T cells, including sensitivity, specificity, positive likelihood ratio (PLR), negative likelihood ratio (NLR), positive predictive value (PPV), and negative predictive value (NPV), are presented in Table 3. It was noted that the above diagnostic measures of IL-27^+^ CD4^+^ T cells were better than their corresponding counterparts of IL-27^+^ CD8^+^ T cells, respectively.

Since IL-27 and ADA have been shown to be helpful in diagnosing TPE^5^,^6^,^7^,^8^, we presented the diagnostic performance of IL-27 and ADA in Table 3, too. Based on the direct comparisons of sensitivity, specificity, PLR, NLR, PPV, and NPV, we noted that the accuracy of IL-27^+^ CD4^+^ T cells was not as good as that of IL-27, and was similar to that of ADA; and that the accuracy of IL-27^+^ CD8^+^ T cells was inferior to that of IL-27 or ADA.

## Discussion

Tuberculosis accounts for millions of active disease cases and deaths in both developed and developing countries. Pulmonary infection with *Mycobacterium tuberculosis* is the most common form of tuberculosis, TPE remains a frequent form of extrapulmonary tuberculosis^9^,^10^. In 2010, China still had an estimated 1 million new tuberculosis cases, accounting for 11% of global tuberculosis incidence, although the prevalence of smear-positive tuberculosis decreased from 170 (95% confidence interval, 166–174) patients to 59 (49–72) patients per 100,000 Chinese population from 1990 to 2010^11^. Moreover, 34.2% (30.9–37.6%) of the new cases of tuberculosis and 54.5% (49.6–59.4%) of the previously treated cases were resistant to at least one of the first-line anti-tuberculosis drugs; 5.7% (4.5–7.0%) of new cases and 25.6% (21.5–29.8%) of previously treated cases were multidrug-resistant tuberculosis^12^. Therefore, tuberculosis continues to be one of the most common causes of mortality and morbidity due to infectious cause in China. Our recent unpublished data showed that during the past 3-year period in a 1600-bed general hospital in China, the cause of pleural effusions in 10.7% (165/1,541) of patients admitted to our hospital was tuberculosis.

Making the diagnosis of with definite etiology, especially the differential diagnosis of TPE and MPE, is extremely difficult and continues to pose clinical challenges, since their similarly clinical and laboratory manifestations and sometimes even lack of pathological or etiological evidence. Sometimes, differential diagnosis of TPE from non-TPEs mandates more invasive procedures like thoracoscopy or thoracotomy^10^,^13^. However, pleural biopsy is invasive, operator-dependent, and technically difficult. Therefore, newer rapid tests and biomarkers have been evaluated^14^. To our best knowledge, the current study was the first one to investigate the diagnostic value of IL-27^+^ CD4^+^ and IL-27^+^ CD8^+^ T cells in discriminating TPE from non-TPEs.

The presence of *Mycobacterium tuberculosis* antigens in pleural space elicits an intense immune response, initiated by macrophages and neutrophils^15^,^16^, followed by Th1 cells^17^,^18^, and resulting in a lymphocyte-predominant exudative effusion. It has been documented that besides Th1 cells, the other Th cell subsets, including regulatory T cells, Th17 cells, Th9 cells, and Th22 cells play important immune regulatory roles in the pathogenesis of TPE^19^. Initial studies with IL-27 receptor deficient mice indicated that IL-27 promotes the generation of Th1-cell responses^20^,^21^. IL-27 regulates various immune diseases through its dual proinflammatory and anti-inflammatory effects on immune responses^22^. Recently, we have demonstrated that human IL-27^+^ CD4^+^ T cells in TPE may represent a distinct Th cell subset with unique expression profiles of transcription factors and proinflammatory cytokines^23^. Moreover, these pleural IL-27^+^ CD4^+^ T cells play immune regulatory roles in TPE by affecting epithelial-mesenchymal transition, wound healing, proliferation, and apoptosis of pleural mesothelial cells via STAT3 signaling-dependent mechanisms^23^,^24^.

Consistent with our previous findings^5^, we once again confirmed in the current study that the numbers of IL-27^+^ CD4^+^ and IL-27^+^ CD8^+^ T cells represented the higher values in TPE, showing a significant increase in comparison with those in MPE. Using ROC curve analysis, we found that AUC was 0.975 for IL-27^+^ CD4^+^ T cells and 0.937 for IL-27^+^ CD8^+^ T cells to diagnose TPE. With the optimal cutoff values, sensitivities/specificities of IL-27^+^ CD4^+^ and IL-27^+^ CD8^+^ T cells were 94.3%/93.5% and 80.0%/93.5%, respectively. These data indicated that both IL-27^+^ CD4^+^ and IL-27^+^ CD8^+^ T cells in pleural fluid are helpful biomarkers in differential diagnosing TPE, and that the overall diagnostic performance of IL-27^+^ CD4^+^ T cells is better than that of IL-27^+^ CD8^+^ T cells.

Because likelihood ratios are also clinically meaningful^25^, we also presented both PLR and NLR as our measures of diagnostic accuracy. A PLR value of 14.46 for IL-27^+^ CD4^+^ T cells suggests that patients with TPE have an about 14-fold higher chance of being positive IL-27^+^ CD4^+^ T cell results compared with patients without TPE. This probability would be considered high enough to begin or to continue anti-tuberculosis treatment of TPE, especially in the case of the absence of any malignant evidence. On the other hand, a NLR value of 0.06 suggests that if IL-27^+^ CD4^+^ T cell determination result is negative, the probability that this patient has TPE is 6%, which is acceptable for ruling out TPE. We also noted that PLR and NLR for IL-27^+^ CD8^+^ T cells were 12.27 and 0.21, respectively, indicating that negative results of IL-27^+^ CD8^+^ T cells are not low enough to rule out TPE. These data confirmed that as diagnostic parameter for TPE, IL-27^+^ CD4^+^ T cells are better than IL-27^+^ CD8^+^ T cells, and IL-27^+^ CD8^+^ T cell determination should not be used alone as a justification to deny or to discontinue anti-tuberculosis therapy.

The PPV and NPV are the proportions of positive and negative results in diagnostic tests that are true positive and true negative results, respectively. The PPV and NPV are not intrinsic to the test; they depend also on the prevalence^26^. A PPV of 91.7% for IL-27^+^ CD4^+^ T cells means that 9.3% of the positive IL-27^+^ CD4^+^ T cell results are false positives; while a NPV of 95.6% means that 4.4% of the negative IL-27^+^ CD4^+^ T cell results are false negatives. Although not perfect, such predictive values should be accepted for ruling in or ruling out TPE in clinical practice, especially in high-tuberculous-incidence areas. In addition, both PPV and NPV of IL-27^+^ CD4^+^ T cells were also higher than those of IL-27^+^ CD8^+^ T cells, which showed a lower but still quite high accuracy.

In conclusion, our current data showed that the numbers of IL-27^+^ CD4^+^ and IL-27^+^ CD8^+^ T cells are increased in TPE compared with MPE; they can serve as differential diagnostic indicators for TPE from MPE. The diagnostic performance of IL-27^+^ CD4^+^ T cells is significantly more accurate than that of IL-27^+^ CD8^+^ T cells.

## Methods

### Subjects

The study protocol was approved by the Institutional Review Board for human studies of Beijing Chao-Yang Hospital, Beijing, China; and informed written consent was obtained from all subjects. The study was carried out in accordance with the approved guideline. From April, 2014 through March, 2015, the consecutive patients with pleural effusions of unknown causes were admitted to the Department of Respiratory and Critical Care Medicine of Beijing Chao-Yang Hospital for diagnostic investigation.

The patients were included subsequently if the examinations of pleural fluid and/or biopsy specimens established a definite diagnosis of TPE or MPE. Patients were excluded if they had received any invasive procedures directed into the pleural cavity or if they had suffered chest trauma within 3 months prior to hospitalization. At the time of sample collection, none of the patients had received any anti-tuberculosis therapy, anti-cancer treatment, corticosteroids, or other nonsteroid anti-inflammatory drugs.

Thirty-five anti-HIV Ab negative patients (age range: 16 to 66 yr) were proven to have TPE, and the diagnosis of TPE was established by the presence of *Mycobacterium tuberculosis* in biopsy specimen, or by demonstration of caseating granulomas or epithelioid cell granulomas in pleural tissue with no evidence of other granulomatous diseases.

MPE was collected from 46 patients (age range: 24 to 82 yr) with newly diagnosed MPE. A diagnosis of MPE was established by demonstration of malignant cells in pleural fluid and/or on pleural biopsy specimen. Histologically, 33 cases were adenocarcinoma, 6 were squamous cell carcinoma, 3 were small cell lung cancer, and 4 were pleural mesothelioma.

Transudative effusion, parapneumonic effusion and empyema were not included in the present study, since it was not possible to isolate sufficient mononuclear cells for performing flow cytometry.

### Sample collection and processing

The pleural fluid (100 mL) was collected in heparin-treated tubes from each subject, using a standard thoracocentesis technique within 24 h after hospitalization. Pleural fluid specimens were immersed in ice immediately and were then centrifuged at 1,200 g for 5 min. Venous blood (5 mL) was drawn simultaneously. Mononuclear cells from pleural fluid and blood were isolated by Ficoll-Hypaque gradient centrifugation (Pharmacia, Uppsala, Sweden) within 1 h.

At the same time, all pleural fluid samples were immediately analyzed for total and differential cell counts, protein, lactate dehydrogenase, glucose, cytology, and bacterial examination. In addition, the cell-free supernatants of pleural fluid were frozen at –80 °C immediately after centrifuge for later determining concentrations of IL-27, and ADA.

### Flow cytometry

The intracellular expression of IL-27 on CD4^+^ and CD8^+^ T cells were determined by flow cytometry after surface or intracellular staining with anti-human-specific Abs conjugated with FITC, phycoerythrin, PerCP-cy5.5, or eFluor 660. These human Abs included anti–CD3, –CD4, –CD8, and –IL-27 mAbs, which were purchased from BD Biosciences (FranklinLakes, NJ) or eBioscience (San Diego, CA). Intracellular staining for IL-27 was performed on mononuclear cells stimulated with phorbol myristate acetate (50 ng/ml; Sigma-AldrichSt. Louis, MO) and ionomycin (1 μM; Sigma-Aldrich) in the presence of GolgiStop (BD Biosciences) for 4 h, and then stained with anti–IL-27 mAb. Appropriate species matched Abs served as isotype control. Flow cytometry was performed on a FACS Canto II (BD Biosciences) and analyzed using BD FCS Diva Software and FCS Epress 4 software (De Novo Software, Los Angeles, CA).

### Measurement of IL-27 and ADA

The concentration of IL-27 in pleural fluid was measured by enzyme linked immunosorbent assay (ELISA) kit according to the manufacturer’s protocol (BioLegend Inc., San Diego, CA, USA). The minimum detectable concentration of IL-27 was 11.0 ng/L. ADA activity was determined using the colorimetric method kit following the manufacturer’s instructions (Zhicheng Biological technology Co. Ltd., Shanghai, China). All samples were assayed in duplicate.

### Statistical analysis

Data were expressed as mean ± SD. Parametric tests were used since IL-27^+^ T cell data were normally distributed as determined by a normality test. Comparisons of data between different groups were performed using Student’s *t* test. Comparisons of IL-27^+^ T cells in pleural fluid and in the corresponding blood were made using paired *t* test. Receiver operating characteristic (ROC) curve analyses were used to evaluate the capacity of IL-27^+^ T cells to differentiate TPE from MPE^27^,^28^. The area under the ROC curve (AUC) was calculated, and 95% confidence intervals (CIs) were used to test the hypothesis that the AUC is 0.5^28^,^29^. Analysis was done using SPSS version 16.0 Statistical Software (Chicago, IL, USA), a p-value < 0.05 was considered as statistically significant.

## Additional Information

**How to cite this article**: Liu, Y.-L. *et al.* Determination of Interleukin 27-Producing CD4^+^ and CD8^+^ T Cells for The Differentiation Between Tuberculous and Malignant Pleural Effusions. *Sci. Rep.* **6**, 19424; doi: 10.1038/srep19424 (2016).

## Figures and Tables

**Table 1:** Biochemical and cytological characteristics in pleural effusions.

	TPE	MPE	p value†
n	35	46	
Sex, male/female, n	19/16	27/19	
Age, yr	40.6 ± 16.9	52.0 ± 15.3	0.002
Protein, g/L	47.4 ± 12.3	44.5 ± 12.3	0.305
Glucose, mmol/L	5.21 ± 1.28	5.10 ± 1.16	0.697
Lactate dehydrogenase, IU/L	566.5 ± 345.4	632.1 ± 332.7	0.390
Total cell counts, ×10^9^/L	2.15 ± 0.84	1.74 ± 0.86	0.038
Differential cell counts, %			
Lymphocytes	77.6 ± 7.3	58.4 ± 12.1	< 0.001
Neutrophils	7.8 ± 4.9	12.9 ± 4.0	< 0.001
Macrophages	12.9 ± 4.9	18.4 ± 11.8	0.012
Mesothelial cells	1.7 ± 1.2	6.2 ± 4.0	< 0.001
Malignant cells	–	4.1 ± 4.4	

Values are presented as mean ± SD. TPE = tuberculous pleural effusion, MPE = malignant pleural effusion.

^†^Comparisons of data between TPE and MPE were performed using Student’s *t* test.

**Table 2:** Numbers of IL-27^+^ CD4^+^ and IL-27^+^ CD8^+^ T cells in pleural effusions and blood.

	TPE	MPE	p value†
n	35	46	
Pleural effusion			
IL-27^+^ CD4^+^ T cells, %	53.2 ± 10.3‡	28.3 ± 6.4	< 0.001
IL-27^+^ CD8^+^ T cells, %	68.5 ± 9.7‡	46.7 ± 10.1§	< 0.001
Blood			
IL-27^+^ CD4^+^ T cells, %	29.8 ± 4.6	28.7 ± 5.2	0.630
IL-27^+^ CD8^+^ T cells, %	44.3 ± 5.8	43.6 ± 5.2	0.367

Values are presented as mean ± SD. TPE = tuberculous pleural effusion, MPE = malignant pleural effusion.

^†^Comparisons of data between TPE and MPE were performed using Student’s *t* test.

^‡^p < 0.001;

^§^p < 0.05 comparisons of data in pleural fluid and in corresponding blood were made using paired *t* test.

**Table 3:** Diagnostic performance of IL-27^+^ cells in differentiating tuberculous from non-tuberculous pleural effusions.

	Cutoff	Sensitivity (%)	Specificity (%)	PLR	NLR	PPV (%)	NPV (%)
		(95% CI)	(95% CI)	(95% CI)	(95% CI)	(95% CI)	(95% CI)
IL-27^+^ CD4^+^ T cells	37.15%	94.3	93.5	14.46	0.06	91.7	95.6
		(80.8 to 99.3)	(82.1 to 98.6)	(4.8 to 43.3)	(0.02 to 0.20)	(77.3 to 98.3)	(84.9 to 99.5)
IL-27^+^ CD8^+^ T cells	59.75%	80.0	93.5	12.27	0.21	90.3	86.0
		(63.1 to 93.5)	(82.1 to 98.6)	(4.10 to 37.2)	(0.10 to 0.40)	(73.9 to 98.0)	(73.1 to 94.2)
IL-27	921.5 ng/L	95.1	97.4	23.9	0.05	96.6	92.6
		(81.2 to 99.4)	(86.6 to 99.1)	(11.8 to 52.1)	(0.02 to 0.24)	(87.5 to 98.5)	(84.2 to 95.7)
ADA	23.5 U/L	90.2	94.6	22.4	0.06	95.4	91.3
		(75.3 to 94.2)	(82.3 to 97.6)	(10.8 to 54.2)	(0.02 to 0.27)	(83.5 to 97.7)	(82.4 to 95.1)

PLR = positive likelihood ratio, NLR = negative likelihood ratio, PPV = positive predictive value, NPV = negative predictive value, ADA = adenosine deaminase.
